# Perceived risk, anxiety, and behavioural responses of the general public during the early phase of the Influenza A (H1N1) pandemic in the Netherlands: results of three consecutive online surveys

**DOI:** 10.1186/1471-2458-11-2

**Published:** 2011-01-03

**Authors:** Marloes Bults, Desirée JMA Beaujean, Onno de Zwart, Gerjo Kok, Pepijn van Empelen, Jim E van Steenbergen, Jan Hendrik Richardus, Hélène ACM Voeten

**Affiliations:** 1 Municipal Public Health Service Rotterdam-Rijnmond, P.O. Box 70032, 3000 LP Rotterdam, The Netherlands; 2 National Institute of Public Health and the Environment, Centre for Infectious Disease Control, P.O. Box 1, 3720 BA Bilthoven, The Netherlands; 3 Maastricht University, Department of Work & Social Psychology, P.O. Box 616, 6200 MD Maastricht, The Netherlands; 4 Erasmus MC, University Medical Center Rotterdam, Department of Public Health, P.O. Box 2040, 3000 CA Rotterdam, The Netherlands

## Abstract

**Background:**

Research into risk perception and behavioural responses in case of emerging infectious diseases is still relatively new. The aim of this study was to examine perceptions and behaviours of the general public during the early phase of the Influenza A (H1N1) pandemic in the Netherlands.

**Methods:**

Two cross-sectional and one follow-up online survey (survey 1, 30 April-4 May; survey 2, 15-19 June; survey 3, 11-20 August 2009). Adults aged 18 years and above participating in a representative Internet panel were invited (survey 1, n = 456; survey 2, n = 478; follow-up survey 3, n = 934). Main outcome measures were 1) time trends in risk perception, feelings of anxiety, and behavioural responses (survey 1-3) and 2) factors associated with taking preventive measures and strong intention to comply with government-advised preventive measures in the future (survey 3).

**Results:**

Between May and August 2009, the level of knowledge regarding Influenza A (H1N1) increased, while perceived severity of the new flu, perceived self-efficacy, and intention to comply with preventive measures decreased. The perceived reliability of information from the government decreased from May to August (62% versus 45%). Feelings of anxiety decreased from May to June, and remained stable afterwards. From June to August 2009, perceived vulnerability increased and more respondents took preventive measures (14% versus 38%). Taking preventive measures was associated with no children in the household, high anxiety, high self-efficacy, more agreement with statements on avoidance, and paying much attention to media information regarding Influenza A (H1N1). Having a strong intention to comply with government-advised preventive measures in the future was associated with higher age, high perceived severity, high anxiety, high perceived efficacy of measures, high self-efficacy, and finding governmental information to be reliable.

**Conclusions:**

Decreasing trends over time in perceived severity and anxiety are consistent with the reality: the clinical picture of influenza turned out to be mild in course of time. Although (inter)national health authorities initially overestimated the case fatality rate, the public stayed calm and remained to have a relatively high intention to comply with preventive measures.

## Background

At the end of April 2009, an outbreak of a new Influenza A (H1N1) virus occurred in Mexico and the United States, spreading rapidly to other countries worldwide. The Influenza A (H1N1) virus has became the dominant influenza strain in most parts of the world. Up to January 2010, around 14000 deaths related to Influenza A (H1N1) were reported worldwide [1]. The virus can cause very severe and fatal illness, but the majority of patients experience mild symptoms comparable to the common seasonal influenza. Important differences with the seasonal flu exist. For example, most severe cases and deaths have occurred in adults under 50 years of age, and severe respiratory failure has been reported more frequently in young and healthy persons [2]. When the World Health Organisation (WHO) raised the pandemic alert to phase 6, the focus shifted towards delaying viral spread through population-based measures, such as hand and respiratory hygiene, and voluntary isolation of symptomatic persons [3-5].

In the Netherlands, a new vaccine against the Influenza A (H1N1) virus became available for specific risk groups in November 2009 [6]. Nevertheless, during the 2009 Influenza A (H1N1) pandemic, behavioural responses of the general public were very important in limiting spread of the virus. Compliance with preventive measures, such as non-medical interventions, antiviral treatment, and vaccination, is dependent upon the willingness and ability of the general public. Compliance with preventive measures is not self-evident. During the SARS epidemic in 2003, the use of face masks was low among adults in Hong Kong and air travellers with influenza-like symptoms [7,8]. In the Netherlands, during an outbreak of avian Influenza among poultry in 2003, large groups of the population did not adhere to personal protective measures or instructions regarding prophylaxis [9].

Surveillance of perceptions and behavioural responses of the general public during pandemics provides useful information for health risk communication and achieving successful changes in public behaviour [10,11]. In recent years, a number of studies have been published on risk perception and public responses in case of a pandemic influenza [12-19]. These studies were conducted prior to the occurrence of the 2009 Influenza A (H1N1) pandemic, in times when pandemic influenza was not regarded as a high threat and information was based on hypothetical scenarios. During the 2009 influenza pandemic a number of studies have been conducted among the general public on risk perception of the Influenza A (H1N1) and intention to take preventive measures [20-22]. These studies consisted of a single, cross-sectional survey. In the present study we aimed to examine perceived risk, feelings of anxiety, and behavioural responses of the Dutch general public related to the outbreak of Influenza A (H1N1) over a period with changing risks and publicity. The first objective of this study was to identify trends over time in risk perception, feelings of anxiety, and behavioural responses (survey 1-3). The second objective was to assess factors significantly associated with taking preventive measures and strong intention to comply with government-advised preventive measures in the future (survey 3).

## Methods

### Timing of the three surveys related to the course of the Influenza A (H1N1) outbreak

The first survey started on 30 April 2009, when the first case of Influenza A (H1N1) was confirmed in the Netherlands. At that time there were 27 confirmed Influenza A (H1N1) cases in eight different European Union (EU) countries. The first survey ended on 4 May, when the number of cases in the EU had increased to more than 100, including 15 human-to-human transmissions [23,24]. The second survey started on 15 June 2009, when there was sustained transmission of the Influenza A (H1N1) virus in several countries and the WHO raised the pandemic alert status to phase 6, characterized by human-to-human spread and community-level outbreaks in more than one WHO region. At that time, there were confirmed cases in 82 countries, including 167 deaths. In the Netherlands, the number of confirmed cases had increased to 60. The second data collection period ended on 19 June; when there were more than 200 deaths worldwide [25,26]. The follow-up survey took place from 11 to 20 August 2009, when the Netherlands counted 1021 confirmed cases, including the first fatal case [27]. On 20 August, the total number of reported and confirmed pandemic influenza cases in the world was 24,8941, including 2430 deaths [27,28].

### Participants

At three different time points, an online survey was filled out by a representative Internet panel, named the Flycatcher panel http://www.flycatcher.eu. This panel consists of people from the Dutch general public who volunteer to participate in online questionnaire surveys. The Flycatcher panel consists of 20,000 members. The distribution of demographic variables (gender, age, region, and level of education) of the panel members is comparable to the general Dutch population. The panel meets high quality requirements and is ISO-certified. For the first and second survey, independent random samples were drawn of approximately 1000 panel members aged 18 years and older. All respondents of the first and second survey were invited to participate in the third (follow-up) survey. Panel members who participated in the first or second survey but did not respond to the follow-up survey (n = 255) were excluded from further analyses. Sampled panel members were invited to participate in this study by sending an email with an Internet link. The surveys were online for a period ranging from 5 to 10 days. Panel members received 1.50 Euro in credits for completion of the survey, which could be exchanged for gift vouchers.

The nature of this general Internet-based survey amongst healthy volunteers from the general population does not require formal medical ethical approval according to the Dutch law [29].

### Online questionnaire

An online questionnaire was developed based on an existing questionnaire used in studies on risk perception and precautionary behaviours of the general public during outbreaks of SARS [30] and avian Influenza [31]. The questionnaire was based on an integrated model to explain health behaviour, including constructs from the Protection Motivation Theory (PMT) [32] and the Health Belief Model (HBM) [33]. These theories were applied because risk perception is one of the central constructs. Risk perception is specified as a combination of perceived severity (a person's belief on how serious contracting the illness would be for him/her) and perceived vulnerability (a person's perception of the chance that he/she will contract the disease). Furthermore, the PMT has two other key constructs besides risk perception, namely response efficacy (a person's belief in the effectiveness of the preventive measure) and self efficacy (a person's level of confidence in his/her ability to perform the preventive measure). The PMT states that a high risk perception will only lead to preventive behaviour if response efficacy and self-efficacy are also high. To examine perceived risk and factors associated with taking preventive measures during the 2009 influenza pandemic we included the following constructs: perceived severity of and vulnerability to Influenza A (H1N1), perceived efficacy of preventive measures, and a persons' ability (self-efficacy) and intention to take measures. Participants were asked about preventive measures against the new flu, namely: 'avoiding crowded places'; 'practicing better hygiene (washing hands more frequent, using tissues when coughing or sneezing)'; 'avoiding persons with Influenza A (H1N1)'; 'wearing face mask'; 'seeking medical advice with the onset of flu symptoms'; 'taking antiviral medication (i.e. Tamiflu)'; and 'staying home from school or work'. In the second and third surveys an additional measure was included: 'getting vaccinated with a new vaccine'. Questions about feelings of anxiety for Influenza A (H1N1) were also added [34]. Maladaptive responses are behaviours which does not protect one's health. Maladaptive responses may result in a lack of following advice from public health authorities. In the second and third surveys maladaptive response items were included and phrased as statements on underestimation, fatalism, and avoidance. The questionnaire concluded with items on amount of information received on Influenza A (H1N1), attention paid to the information, reliability and sufficiency of information provided by the government, information needs, and preferences for ways of communication during the further course of the Influenza pandemic. Knowledge was examined by statements concerning modes of transmission, infectiousness, feasibility of symptoms, and fatality of Influenza A (H1N1). The questionnaire was similar across the three survey rounds (Additional file 1). For knowledge, a summary score was created based on the number of correct answers and dichotomized as 0 (< 4 items correct) or 1 (≥4 items correct). For all other constructs with 2 or more items, Cronbach's alpha was calculated. The Cronbach's alpha of the constructs ranged from 0.6 to 0.9. Therefore, a summary score was formulated by adding up the scores of the individual items, and dichotomized on the median.

### Analysis

Time trends were analyzed using the Chi-square test for differences between surveys 1 (May 2009) and 2 (June 2009). Survey 3 of August 2009 was divided into 3.1 (follow-up of survey 1), and 3.2 (follow-up of survey 2); the Mc-Nemar test was used for analyzing differences between surveys 1 and 3.1 and between 2 and 3.2. Univariate and multivariate logistic regression analyses were performed to identify factors significantly associated with taking one or more preventive measures and strong intention to comply with government-advised preventive measures in the future. For the regression analyses we used data from survey 3 (August 2009), when a substantial amount of people took preventive measures (resp. 40%) compared to survey 1 and 2 (resp. 11% and 14%). For the multivariate regression analyses, all factors with a p-value <0.1 in the univariate analysis were entered in the multivariate model, and removed one-by-one (starting with the most insignificant one etc.) until only statistically significant predictors (p < 0.05) remained.

## Results

### Response rates and demographic characteristics

During the first survey in May 2009, 973 panel members were invited and 59% completed the online questionnaire. During the second survey in June 2009, 981 panel members were invited with a response rate of 63%. Of the 1192 respondents from the first two rounds who were invited for the follow-up survey in August, 79% completed the questionnaire.

Demographic characteristics of respondents are listed in Table 1. Overall, there were no significant differences between surveys. Focusing on survey 3, mean age was 51 years (range 19-89 years) and most respondents (92%) were of Dutch origin. Thirty-eight percent had a lower education (i.e. primary education, lower general or lower vocational education or less), 36% an intermediate (i.e. secondary general or vocational education), and 26% a higher education (i.e. higher professional education or university). The majority of respondents were employed. About three quarters were married or cohabitating and in 27% of the households there were one or more children under 18 years. Compared to the general Dutch population (Table 1), the respondents were older, more often of Dutch origin, and more often unemployed/retired.

**Table 1:** Demographic characteristics of respondents, survey 1, 2 and 3

Characteristics	Survey 1	Survey 2	Survey 3 follow-up	Data Statistics NL
	30 April-4 May	15-19 June	11-20 August	1-1-2009
	(n = 456)	(n = 478)	(n = 934)	
**Sex**				
Male	52%	52%	52%	50%
Female	48%	49%	48%	51%
**Age**				
18-29 years	13%	12%	12%	18%
30-49 years	33%	40%	36%	37%
Above 50 years	55%	49%	52%	44%
**Ethnicity**				
Dutch	90%	92%	92%	80%
Non-dutch	10%	8%	8%	20%
**Education**				
Low	40%	39%	38%	33%
Intermediate	38%	38%	36%	41%
High	22%	23%	26%	25%
**Employment status**				
Employed	-	61%	57%	76%
Unemployed/Retired	-	40%	43%	24%
**Marital status**				
Single	-	17%	19%	
Married/Cohabitating	-	76%	73%	
Divorced/Widowed	-	7%	7%	
**Children < 18 years in household**				
Yes	-	27%	27%	
No	-	73%	73%	

'-' data not collected in survey 1.

### Time-trends in perceived risk, feelings of anxiety, and behavioural responses

The level of knowledge regarding Influenza A (H1N1) was generally high (Table 2). The percentage of respondents who answered 4 or more out of 6 items correctly increased significantly over time during the survey period, from 88% in May to 95% in August 2009 (for the survey in August, we refer to the results of survey 3.2). Only knowledge regarding the availability of a vaccine (which was not available before November 2009) decreased.

**Table 2:** Trends over time in risk perception, anxiety and behavioural responses

	Survey 1 30 April-4 May (n = 456)	Survey 2 15-19 June (n = 478)	P-value^†^ survey 1 vs. 2	Survey 3.1 follow-up^τ^ 11-20 August (n = 456)	P-value^‡^ survey 1 vs. 3.1	Survey 3.2 follow-up^π^ 11-20 August (n = 478)	P-value^‡^ survey 2 vs. 3.2	Time trend	
								1-2	2-3
**Knowledge**									
1. The new flu is caused by a new influenza virus (correct)	74%	79%	ns	84%	< 0.001	86%	< 0.001	ns	+
2. A vaccine is available against the new flu (incorrect)^§^	50%	47%	ns	37%	< 0.001	36%	< 0.001	ns	—
3. The new flu can be transmitted by human-to-human contact (correct)	97%	98%	ns	98%	ns	99%	ns	ns	ns
4. People died from the new flu (correct)	97%	97%	ns	99%	0.03	99%	ns	ns	ns
5. The new flu can be transmitted through eating pork (incorrect)	91%	90%	ns	95%	0.004	94%	0.01	ns	+
6. Symptoms of the new flu are visible (incorrect)	81%	81%	ns	90%	< 0.001	87%	< 0.001	ns	+
7. A flu pandemic occurs once in 10-50 years (correct)	-	56%	-	50%	-	60%	ns		ns
Summary score (4 or more correctly answered)	88%	92%	0.02	96%	< 0.001	95%	0.05	+	+
							
**Perceived severity (scale 1-5)**									
1. Severity of the new flu (score 4-5, severe-very severe)	80%	67%	< 0.001	43%	< 0.001	39%	< 0.001	—	—
2. Severity of getting the new flu coming year (score 4-5, severe-very severe)	70%	61%	0.002	39%	< 0.001	39%	< 0.001	—	—
3. The new flu is very harmful for my health (score 4-5, mostly-totally agree)	-	49%	-	31%	-	27%	< 0.001		—
Summary score items 1-2 (high) - Chronbach alpha 0.8	64%	53%	< 0.001	29%	< 0.001	29%	< 0.001	—	—
							
**Perceived vulnerability (scale 1-5)**									
1. Perceived susceptibility (score 4-5, quite-very susceptible)	18%	22%	ns	26%	0.02	30%	0.003	ns	+
2. Perceived chance of getting infected next year (score 4-5, likely-very likely)	5%	5%	ns	15%	< 0.001	15%	< 0.001	ns	+
3. Perceived chance of getting infected compared to others (score 4-5, more-much more)	6%	6%	ns	9%	ns	12%	< 0.001	ns	+
Summary score (high) - Chronbach alpha 0.6	15%	16%	ns	29%	< 0.001	31%	< 0.001	ns	+
							
**Perceived anxiety (scale 1-5)**									
1. Worried about the new flu (score 4-5, worried-very worried)	36%	19%	< 0.001	16%	< 0.001	14%	0.02	—	—
2. Fear for the new flu (score 4-5, scared-very scared)	16%	8%	< 0.001	6%	< 0.001	4%	0.009	—	—
3. Thinking about the new flu (score 4-5, often-very often)	27%	9%	< 0.001	12%	< 0.001	15%	0.003	—	+
Summary score (high) - Chronbach alpha 0.8	61%	40%	< 0.001	39%	< 0.001	36%	ns	—	ns
							
**Perceived efficacy (scale 1-5; certainly not-certainly)**									
1. Keep away from crowded places (score 4-5)	55%	47%	0.01	58%	ns	54%	0.01	—	+
2. Practice better hygiene (score 4-5)	80%	75%	ns	89%	< 0.001	89%	< 0.001	ns	+
3. Avoid regions/persons^¥^ with new flu (score 4-5)	82%	73%	0.001	74%	0.002	73%	ns	—	ns
4. Wear face mask (score 4-5)	34%	31%	ns	22%	< 0.001	25%	0.009	ns	—
5. Seek medical advice with the onset of flu symptoms (score 4-5)	78%	72%	0.03	70%	< 0.001	66%	0.03	—	—
6. Take antiviral medication (score 4-5)	37%	46%	0.005	40%	ns	39%	0.01	+	—
7. Stay home from school or work (score 4-5)	18%	21%	ns	33%	< 0.001	31%	< 0.001	ns	+
8. Get a new vaccine against the new flu (score 4-5)	-	54%	-	49%	-	53%	ns		ns
Summary score items 1-7 (high) - Chronbach alpha 0.7	50%	38%	< 0.001	50%	ns	50%	< 0.001	—	+
							
Perceived self-efficacy* **(scale 1-5; certainly not-certainly)**									
1. Keep away from crowded places (score 4-5)	67%	61%	ns	56%	< 0.001	50%	< 0.001	ns	—
2. Practice better hygiene (score 4-5)	91%	88%	ns	89%	ns	88%	ns	ns	ns
3. Avoid regions/persons^¥^ with new flu (score 4-5)	89%	78%	< 0.001	64%	< 0.001	66%	< 0.001	—	—
4. Wear face mask (score 4-5)	71%	60%	< 0.001	50%	< 0.001	47%	< 0.001	—	—
5. Seek medical advice with the onset of flu symptoms (score 4-5)	91%	87%	0.05	87%	0.02	86%	ns	—	ns
6. Take antiviral medication (score 4-5)	80%	80%	ns	71%	0.001	70%	< 0.001	ns	—
7. Stay home from school or work (score 4-5)	56%	50%	ns	52%	ns	50%	ns	ns	ns
8. Get a new vaccine against the new flu (score 4-5)	-	79%	-	69%	-	70%	< 0.001		—
Summary score items 1-7 (high) - Chronbach alpha 0.9	55%	43%	< 0.001	38%	< 0.001	35%	0.001	—	—
							
Intention* **(scale 1-5; certainly not-certainly)**									
1. Keep away from crowded places (score 4-5)	76%	66%	0.001	62%	< 0.001	59%	0.001	—	—
2. Practice better hygiene (score 4-5)	93%	89%	ns	91%	ns	89%	ns	ns	ns
3. Avoid regions/persons^¥^ with new flu (score 4-5)	89%	81%	0.001	71%	< 0.001	72%	< 0.001	—	—
4. Wear face mask (score 4-5)	70%	57%	< 0.001	46%	< 0.001	44%	< 0.001	—	—
5. Seek medical advice with the onset of flu symptoms (score 4-5)	91%	89%	ns	84%	< 0.001	84%	0.01	ns	—
6. Take antiviral medication (score 4-5)	82%	76%	0.02	66%	< 0.001	65%	< 0.001	—	—
7. Stay home from school or work (score 4-5)	61%	53%	0.01	56%	ns	50%	ns	—	ns
8. Get a new vaccine against the new flu^§^ (score 4-5)	-	77%	-	67%	-	63%	0.001		—
Summary score items 1-7 (high) - Chronbach alpha 0.9	60%	48%	< 0.001	41%	< 0.001	41%	0.006	—	—
							
**Maladaptive response (scale 1-5; totally disagree-totally agree)**									
The threat is exaggerated by media and government (score 4-5)	-	35%	-	56%	-	58%	< 0.001		+
It will not be as bad as predicted (score 4-5)	-	28%	-	49%	-	49%	< 0.001		+
Summary score - underestimation statements (high) - Chronbach alpha 0.6	-	20%	-	40%	-	42%	< 0.001		+
There is nothing we can do about it (score 4-5)	-	5%	-	14%	-	16%	< 0.001		+
We will all be completely powerless (score 4-5)	-	7%	-	14%	-	14%	< 0.001		+
We will just have to accept it (score 4-5)	-	24%	-	43%	-	47%	< 0.001		+
Summary score - fatalism statements (score 4-5) - Chronbach alpha 0.6	-	26%	-	48%	-	44%	< 0.001		+
I will move to a place without influenza (score 4-5)	-	2%	-	1%	-	0%	0.04		—
I will stock up and stay indoors (score 4-5)	-	3%	-	2%	-	4%	ns		ns
Summary score - avoidance statements (high) - Chronbach alpha 0.7	-	52%	-	39%	-	38%	< 0.001		—
							
**Information (scale 1-5)**									
Amount of information received (score 4-5, much-very much)	52%	37%	< 0.001	53%	ns	48%	< 0.001	—	+
Attention paid to information received (score 4-5, much-very much)	30%	21%	0.002	21%	< 0.001	23%	ns	—	ns
Is information of the government reliable? (score 4-5, reliable-very reliable)	62%	53%	0.004	48%	< 0.001	45%	0.002	—	—
Is information of the government sufficient? (score 4-5, sufficient-very sufficient)	58%	42%	< 0.001	52%	0.02	47%	ns	—	ns
							
**Measures taken**									
Practiced better hygiene	8%	12%	ns	36%	< 0.001	36%	< 0.001	ns	+
Avoided persons with influenza-like symptoms	-	4%	-	10%	-	9%	< 0.001		+
Avoided crowded places	3%	3%	ns	7%	0.003	8%	< 0.001	ns	+
Cancelled or did not book a holiday to areas with the new flu	0.2%	0.4%	ns	0.9%	ns	1%	ns	ns	ns
Bought face mask	0.4%	1%	ns	0.7%	ns	2%	ns	ns	ns
Bought antiviral medication	0.2%	0.4%	ns	0.4%	ns	2%	ns	ns	ns
Something else	1%	2%	ns	2%	ns	1%	ns	ns	ns
Summary score (any measures taken)	11%	14%	ns	40%	< 0.001	38%	< 0.001	ns	+

vs = versus; ^† ^p-value obtained using Chi^2 ^tests; ^‡ ^p-value obtained using McNemar tests; ^τ ^follow-up of survey 1; ^π ^follow-up of survey 2; '-' data not collected in survey 1;

'+' indicates a significant increase over time; '—' indicates a significant decrease over time; ns = not statistically significant.

^¥ ^In the third survey 'avoiding regions with Influenza A (H1N1)' was changed into 'avoiding persons with influenza like symptoms'.

* Respondents were asked to imagine that governmental health institutes would recommend the preventive behaviour.

^§ ^A vaccine against Influenza A (H1N1) became available in the Netherlands in November 2009.

The percentage of respondents who reported a high perceived severity of Influenza A (H1N1) decreased from 80% in May to 39% in August 2009, whereas a high perceived vulnerability increased from 5% in June to 15% in August (Table 2). Feelings of anxiety decreased from 16% in May to 4% in August reporting being (very) scared for the new flu. The perceived efficacy of preventive measures was highest for practicing better hygiene, avoiding regions with the new flu or persons with influenza-like symptoms, and seeking medical advice with the onset of flu symptoms; the percentage who perceived these measures to be effective ranging from 66% to 89% in August 2009. At the same time respondents felt confident to practice these preventive measures (perceived self-efficacy) ranging from 66% who felt confident to avoid persons with the new flu to 88% who felt confident to practice better hygiene. The intention to comply decreased significantly over the three surveys for four out of seven preventive measures. The highest intention to comply was reported for practicing better hygiene and seeking medical advice, the lowest for staying home from school or work and wearing a face mask. The percentage of respondents who were likely to get vaccinated against Influenza A (H1N1) (when advised by the government) decreased from 77% in June to 63% in August.

Over time, more respondents agreed with the statement that the threat of the new flu was exaggerated by the media or government (35% June, 58% August) and that it would not be as bad as predicted (28% June, 49% August). Also, a larger number of respondents were in agreement with the statement 'we just have to accept it', increasing from 24% in May to 47% in August.

The amount of received information about Influenza A (H1N1) decreased significantly between May and June and increased between June and August 2009, with the percentage of respondents who received (very) much information increasing from 37% to 48%. Information from the government was found less reliable over time; 62% found the information of the government reliable in May; in August 2009 this value decreased to 45%. In August, 70% reported a need for more information, mainly regarding details on the symptoms of Influenza A (H1N1) (30%), how to prevent infection (27%), and how it can be treated (16%) (data not shown). The preferred method for receiving this information was television (47%), Internet (36%), and newspapers (36%). The respondents preferred this information to be given by local or national health institutes or their general practitioner.

There was an increase in the percentage of respondents who had taken any preventive measure between June (14%) and August 2009 (38%). Practicing better hygiene was reported most often, by 36% of the respondents at the last survey. For the specific measures, a significant increase over time was observed for practicing better hygiene (12% in June, 36% in August), avoiding persons with influenza like symptoms (4% in June, 9% in August), and avoiding crowded places (3% in June, 8% in August) (Table 2).

### Factors associated with taking preventive measures and strong intention to comply (survey 3)

Univariate and multivariate logistic regression analyses were performed to identify factors significantly associated with: 1) taking one or more preventive measures and 2) strong intention to comply with government-advised preventive measures in the future (Table 3). In this regression analysis variables of the survey in August (survey 3) were included.

**Table 3:** Predictors of taking preventive measures and strong intention to comply with measures regarding Influenza A (H1N1)

	Taking one or more preventive measures							Strong intention to comply with government-advised preventive measures in the future^†^						
	
	Row %	OR_u_	95% CI	p-value	OR_m_	95% CI	p-value	Row %	OR_u_	95% CI	p-value	OR_m_	95% CI	p-value
	
Demographic characteristics														
Sex														
male	36.9	1.00						45.3	1.00					
female	41.5	1.21	0.93-1.58	0.1	-	-	-	53.0	1.36	1.05-1.76	0.02	-	-	-
Age														
18-29 years	35.1	1.00		ns	-	-	-	28.9	1.00		<0.001	1.00		0.007
30-49 years	36.2	1.05	0.67-1.64					44.2	1.95	1.23-3.08		1.77	0.94-3.35	
above 50 years	42.0	1.34	0.88-2.05					57.1	3.27	2.10-5.10		2.61	1.39-4.90	
Ethnicity														
Dutch	39.1	1.00		ns	-	-	-	49.4	1.00		ns	-	-	-
non-Dutch	39.0	1.00	0.62-1.60					45.5	0.86	0.54-1.37				
Education														
low	37.8	1.00		ns	-	-	-	56.3	1.00		0.002	-	-	-
intermediate	40.2	1.11	0.82-1.50					45.9	0.66	0.49-0.89				
high	39.3	1.07	0.76-1.49					42.6	0.58	0.42-0.80				
Employment status														
employed	38.9	1.00		ns	-	-	-	41.5	1.00					
unemployed/retired	39.4	1.02	0.78-1.33					58.9	2.02	1.55-2.63	<0.001	-	-	-
Marital status														
single	39.4	1.00		ns	-	-	-	41.7	1.00		0.04			
married/cohabited	39.3	0.99	0.71-1.39					50.1	1.40	1.01-1.96				
divorced/widowed	36.2	0.87	0.49-1.55					58.0	1.93	1.10-3.39		-	-	-
Children < 18 yrs in household														
yes	34.0	1.00			1.00			44.8	1.00					
no	40.9	1.35	0.99-1.82	0.06	1.45	1.04-2.00	0.03	50.6	1.27	0.94-1.69	0.1	-	-	-
Knowledge score														
<4	36.5	1.00						47.0	1.00		ns	-	-	-
≥4	41.1	1.22	0.93-1.59	0.1	-	-	-	50.6	1.15	0.89-1.49				
Perceived severity (summary score)														
low severity	34.0	1.00						37.3	1.00			1.00		
high severity	44.5	1.56	1.20-2.03	0.001	-	-	-	61.7	2.71	2.08-3.53	<0.001	1.62	1.07-2.44	0.02
Perceived vulnerability (summary score)														
low vulnerability	35.4	1.00						45.2	1.00					
high vulnerability	47.5	1.66	1.25-2.20	<0.001	-	-	-	57.7	1.66	1.25-2.19	<0.001	-	-	-
Anxiety (summary score)														
low anxiety	30.6	1.00			1.00			39.5	1.00			1.00		
high anxiety	53.1	2.57	1.96-3.38	<0.001	1.93	1.43-2.61	<0.001	64.8	2.81	2.14-3.70	<0.001	2.22	1.44-3.42	<0.001
Perceived efficacy (summary score)														
low efficacy	32.8	1.00						31.0	1.00			1.00		
high efficacy	46.4	1.77	1.36-2.31	<0.001	-	-	-	70.1	5.21	3.94-6.89	<0.001	2.57	1.77-3.74	<0.001
Perceived self-efficacy (summary score)														
low self-efficacy	31.3	1.00			1.00			18.4	1.00			1.00		
high self-efficacy	48.6	2.08	1.59-2.72	<0.001	1.68	1.26-2.22	<0.001	86.3	27.9	19.5-39.8	<0.001	21.53	14.70-31.55	<0.001
Maladaptive response (summary score)														
Underestimation statements														
(fully) disagree/not disagree-agree (1-3)	43.0	1.00						56.5	1.00					
(fully) agree (4-5)	33.4	0.67	0.51-0.87	0.003	-	-	-	38.2	0.48	0.36-0.62	<0.001	-	-	-
Fatalism statements														
(fully) disagree/not disagree-agree (1-3)	43.3	1.00						57.1	1.00					
(fully) agree (4-5)	34.1	0.68	0.52-0.89	0.004	-	-	-	39.5	0.49	0.38-0.64	<0.001	-	-	-
Avoidance statements														
(fully) disagree/not disagree-agree (1-3)	34.6	1.00			1.00			45.7	1.00					
(fully) agree (4-5)	46.2	1.63	1.24-2.13	<0.001	1.43	1.07-1.90	0.02	54.3	1.41	1.08-1.84	0.01	-	-	-
Amount of information received														
nothing/little/some (1-3)	33.2	1.00						46.2	1.00					
much/very much (4-5)	44.8	1.64	1.25-2.13	<0.001	-	-	-	51.8	1.25	0.97-1.62	0.09	-	-	-
Attention paid to the information														
(very) little/some (1-3)	33.1	1.00			1.00			44.4	1.00					
much/very much (4-5)	61.3	3.19	2.31-4.40	<0.001	2.36	1.67-3.33	<0.001	66.7	2.51	1.81-3.47	<0.001	-	-	-
Reliability of governmental information														
not (at all)/little reliable (1-3)	36.0	1.00						39.8	1.00			1.00		
(very) reliable (4-5)	42.7	1.33	1.02-1.73	0.04	-	-	-	59.9	2.26	1.74-2.94	<0.001	1.74	1.19-2.55	0.004
Sufficiency of governmental information														
not (at all)/little sufficient (1-3)	36.8	1.00						46.2	1.00					
(very) sufficient (4-5)	41.5	1.22	0.94-1.59	0.1	-	-	-	52.0	1.26	0.97-1.63	0.08	-	-	-
R^2^					0.14							0.60		

Data from survey 3 were used for the regression analyses (August 2009, n = 934).

OR_u_ : univariate odds ratio; OR_m_ : multivariate odds ratio; ns: not statistically significant.

Included preventive measures, i.e. 1) keep away from crowded places; 2) practice better hygiene; 3) avoid persons with the new flu; 4) wear face mask; 5) seek medical advice with the onset of flu symptoms;

6) take antiviral medication; 7) stay home from school or work; 8) get a new vaccine against the new flu.

Factors univariately associated with taking preventive measures but not significant in the multivariate analysis were: perceived severity, vulnerability, and efficacy of measures; underestimation and fatalism statements; amount of information received, and reliability of governmental information. From multivariate logistic regression analysis, predictors of taking preventive measures were no children in the household (OR 1.45; 95% CI 1.04-2.0), high anxiety (OR 1.93; 95% CI 1.43-2.61), higher level of self-efficacy (OR 1.68; 95% CI 1.26-2.22), more in agreement with statements on avoidance (OR 1.43; 95% CI 1.07-1.90), and paying much attention to the information on Influenza A (H1N1) (OR 2.36; 95% CI 1.67-3.33).

We also took a strong intention to comply with measures in the near future, when advised by the government, as an outcome (dependent) variable in the logistic regression analyses. Factors that were univariately associated but not significant in the multivariate analysis were: gender, level of education, employment status, marital status, perceived vulnerability, underestimation, fatalism, and avoidance statements, and attention paid to the information on Influenza A (H1N1). In the multivariate logistic regression model, predictors of a strong intention to comply were older age (> 50 yrs: OR 2.61; 95% CI 1.39-4.90), higher levels of perceived severity (OR 1.62; 95% 1.07-2.44), feelings of anxiety (OR 2.22; 95% CI 1.44-3.42), believing in the efficacy of measures (OR 2.57; 95% CI 1.77-3.74), self-efficacy (OR 21.53; 95% CI 14.70-31.55), and finding government information to be reliable (OR 1.74; 95% CI 1.19-2.55).

## Discussion

In this population-based study performed in the Netherlands, we found that the level of knowledge regarding Influenza A (H1N1) increased between May and August 2009. At the same time, perceived severity of the new flu, perceived self-efficacy, and intention to comply with preventive measures decreased. The perceived reliability of information from the government also decreased from May to August. Feelings of anxiety decreased from May to June, and remained stable afterwards. From June to August 2009, perceived vulnerability increased and more respondents took preventive measures. Factors associated with taking preventive measures included no children in the household, high anxiety, high self-efficacy, agreeing with avoidance statements, and paying much attention to media information regarding Influenza A (H1N1). Having a strong intention to comply with government-advised preventive measures in the future was associated with older age, high perceived severity, high anxiety, high perceived efficacy of measures, high self-efficacy, and finding governmental information to be reliable.

A clear strength of this study is that data collection took place during the 2009 Influenza A (H1N1) pandemic, in contrast to other studies performed at times when pandemic influenza was not regarded as a high threat and scenarios were based on hypothetical situations [12-19]. Another strength is that this study consists of three repeated survey rounds, enabling analysis of trends over time. This is in contrast to other recent studies, which consisted of a single cross-sectional survey [20-22]. Moreover, we followed-up individuals, guaranteeing that differences between survey rounds were not due to differences between study populations, but represent real trends over time [35]. Finally, we used an online questionnaire, which creates less social desirability bias than personal telephone interviews. The use of an Internet panel led to high response rates: 59%, 63%, and 79% in survey 1, survey 2, and survey 3, respectively. Our study also has several limitations. First, the Internet panel members who responded to our online questionnaire were not fully representative of the general Dutch population. In our study, participants were more likely to be in the older age group (> 50 years) (52% versus 44%), of Dutch ethnicity (92% versus 80%), and unemployed/retired (43% versus 24%). We cross tabulated all the measured constructs by age group (18-49 years/> 50 years), employment status (employed/unemployed) and ethnicity (Dutch/non-Dutch) (data not shown). For these constructs, there were no differences between the Dutch and non-Dutch participants. Among both the older and unemployed the perceived efficacy, self efficacy and intention to comply with measures were significantly higher, and they more agreed with statements on avoidance. Perceived vulnerability and reliability of governmental information were lower among both the older and unemployed. Furthermore, the older age group paid more attention to the information of the government. Among the unemployed the perceived severity was higher and they less agreed with the underestimation statements. This population difference may have led to a substantial bias in the absolute outcomes of Table 2, but only to a small bias in the trends over time or in the predictors of behavioural responses. Second, in the logistic regression analyses we may have lost some power, because we used dichotomized summary scales as predictors. However, we have performed additional regression analyses with the predictors as continuous variables, and found minimal differences (data not shown). Third, the validity of the questionnaire used in this study was not tested through a test-retest design, because the Influenza pandemic was ongoing and thus perceptions were not stable over time. Fourth, no data were obtained from non-responders.

This is the first national study to evaluate perceived risk, feelings of anxiety, and behavioural responses regarding Influenza A (H1N1) among the general public in the Netherlands. There was a decrease over time in perceived severity, anxiety and intention to comply with preventive measures. Initially, representatives of (inter)national health institutes predicted a worse-case scenario with large numbers of fatal cases, based on influenza pandemics in the past and early reports concerning the new Influenza virus [36]. In the following months, media attention decreased considerably, local viral transmission remained relatively limited in the Netherlands, and the Dutch government announced that the pandemic appeared to be mild [37,38]. Decreasing trends over time in perceived severity and anxiety are consistent with the reality: the clinical picture of influenza turned out to be mild in course of time. The decrease in perceived reliability of information from the government was not surprisingly; in the beginning the general public believed the pandemic would be severe as pronounced by the government, but this turned out to be mild. This decrease in perceived reliability of governmental information was not alarming and did not result in more feelings of anxiety or in a lower intention to comply with measures. The increase in perceived vulnerability and number of individuals taking preventive measures may be an effect of the increasing number of Influenza A (H1N1) infected cases, including the first fatal case in The Netherlands in August 2009. Previous studies showed a similar effect. For instance with the inclining phase of the SARS outbreak in 2003, the prevalence of wearing a face mask and adopting better hand hygiene increased dramatically when the number of SARS cases increased [39]. During the current study period, there was no official recommendation from the Dutch government to take preventive measures; the government was in the process of preparing a national information campaign called 'Fight the flu'. This campaign was launched at the end of August 2009, and included announcements on television and a leaflet which was sent to every home in the country providing information about what people can do to prevent themselves and others. So, at the moment of the third data collection period the government had not yet actively informed the general public about preventive measures. For this reason, respondents were not only asked about preventive measures they had taken, but also about their intention to comply with government-advised preventive measures in the near future. People who took preventive measures during this 'pre-phase' of governmental advice were very alert to media information and seemed to be practicing preventive measures based on emotions such as anxiety. This is in line with results of the study conducted by Jones et al. [20] concluding that affective variables, such as self-reported anxiety over the epidemic, mediate the likelihood that respondents engage in protective behaviour. Rubin et al. [21] also found a significant association between anxiety and carrying out recommended behaviours. Similarly, studies on outbreaks of SARS found that anxiety was associated with taking preventive measures [39,40]. To date, there are only few published studies assessing factors that might explain compliance with preventive behaviours in case of an Influenza pandemic. Comparison with these studies is difficult because of differences in phrasing of questionnaire items and methods of analysis. Barr et al. [14] collected baseline data about willingness to comply with vaccination, isolation, and wearing a face mask among Australians during a hypothetical influenza pandemic, and found a higher level of willingness to comply among people with higher levels of threat perception and among those of older age. This is in agreement with our findings, where intention to comply with measures was also associated with older age and high perceived severity.

This is one of the first studies conducted during the course of the Influenza pandemic. Additional studies on risk perception among the public are needed to further understand the field of preventive behaviour as related to control of infectious diseases. Furthermore, these studies need to address emotional aspects such as anxiety, uncertainty, or embarrassment that play a role in decision making. Finally, research regarding the translation of results from the above-suggested studies into risk communication is of utmost importance.

Our study has several implications for health authorities and public health policy. In case of an emerging infectious disease, as Influenza A (H1N1), it is very difficult to predict the further course of the outbreak. It is important that health authorities present a range of scenarios, not only worst-case but also other, more positive, scenarios. In the beginning of an outbreak, there are many uncertainties about the infectiousness and case fatality rate of the disease. Health authorities should not only communicate with the public about 'what is known' (the certainties), but they should also communicate about 'what is not known' (the uncertainties). In course of the outbreak, when more information becomes available, public health authorities should update their messages to achieve effective risk communication. This is essential not only to instruct and motivate the public to take preventive measures, but also to build trust in public health authorities and prevent misconceptions. Besides rational arguments (such as perceived severity and efficacy of measures), emotional aspects like anxiety play a role in decision making concerning preventive behaviour. Health authorities should acknowledge these emotional aspects and take these arguments into account in their risk communication with the general public.

## Conclusion

Decreasing trends over time in perceived severity and anxiety are consistent with the reality: the clinical picture of influenza turned out to be mild in course of time. Although (inter)national health authorities initially overestimated the case fatality rate, the public stayed calm and remained to have a relatively high intention to comply with preventive measures. During future outbreaks of infectious diseases it is important that health authorities present a range of scenarios, not only worst-case but also other, more positive, scenarios. Health authorities should not only communicate with the public about 'what is known' (the certainties), but they should also communicate about 'what is not known' (the uncertainties). In course of the outbreak, when more information becomes available, public health authorities should update their messages to instruct and motivate the public to take preventive measures, to build trust in public health authorities and prevent misconceptions.

## Competing interests

The authors declare that they have no competing interests.

## Authors' contributions

All authors contributed to the study design. MB, DB, and HV played a main role in the data collection process. Data analysis was performed by MB and HV with advice of PvE. MB, DB, OdZ, and HV wrote the first draft of the manuscript; GK, PvE, JvS, and JHR critiqued the manuscript and contributed to further drafts. HV is the guarantor. All authors read and approved the final manuscript.

## Pre-publication history

The pre-publication history for this paper can be accessed here:

http://www.biomedcentral.com/1471-2458/11/2/prepub

## Supplementary Material

Additional file 1 **Survey questions 'Risk perception and behavioural responses of the general public during the Influenza A (H1N1) pandemic in the Netherlands'**. This questionnaire was used across the three survey rounds. Click here for file
