# Supplementary material for: Perceived risk, anxiety, and behavioural responses of the general public during the early phase of the Influenza A (H1N1) pandemic in the Netherlands: results of three consecutive online surveys
Source: BMC Public Health. 2011 Jan 3;11:2. doi: 10.1186/1471-2458-11-2 (PMC3091536; doi:10.1186/1471-2458-11-2)
Supplement: Additional file 1 — Survey questions 'Risk perception and behavioural responses of the general public during the Influenza A (H1N1) pandemic in the Netherlands'. This questionnaire was used across the three survey rounds. [file 1471-2458-11-2-S1.PDF]

Additional file 1
Survey questions 'Risk perception and behavioural responses of the general public during the Influenza A (H1N1) pandemic in the Netherlands'.

We would first ask you to answer some questions about your personal circumstances.

1. **What is your country of birth?**

    - The Netherlands
    - Dutch Antilles/Aruba
    - Belgium
    - Germany
    - Indonesia
    - Morocco
    - Surinam
    - Turkey
    - Other, namely..

2. **Are you employed at the moment?**
    - Yes
    - No

3. **What is your marital status?**
    - Single
    - Cohabitating
    - Married
    - Divorced
    - Widowed

4. **Do you have children (younger than 18 years) in your household?**
    - No
    - 1 child
    - 2 children
    - 3-4 children
    - 5 or more children

This survey is about the Mexican flu.

5. **Below statements are formulated about the Mexican flu. Please indicate whether the following statements are right or false?**

	Right	False	Don't know
- The Mexican flu is caused by a new influenza virus	○	○	○
- A vaccine is available against the Mexican flu	○	○	○
- The Mexican flu can be transmitted by human-to-human contact	○	○	○
- People died from the Mexican flu	○	○	○
- The Mexican flu can be transmitted through eating pork	○	○	○
- Symptoms of the Mexican flu are visible	○	○	○
- A flu pandemic occurs once in the 10-50 years	○	○	○

6. **How severe do you think the Mexican flu is?**
    - Not severe at all
    - Not severe
    - Even
    - Severe
    - Very severe

7. **The Mexican flu is very harmful for my health.**
    - Totally disagree
    - Mostly disagree
    - Don't agree or disagree
    - Mostly agree
    - Totally agree

8. **A number of medical conditions are mentioned below. For each condition, please indicate how awful it would be if you were to be diagnosed with this condition in the next 12 months?**

	Not severe at all	Not severe	Even	Severe	Very severe
Seasonal influenza	○	○	○	○	○
Diabetes	○	○	○	○	○
Heart attack	○	○	○	○	○
Mexican flu	○	○	○	○	○
HIV or AIDS	○	○	○	○	○

9. **Do you think that, in general, you are susceptible to getting the Mexican flu if you take no preventive measures?**
    - Not at all susceptible
    - No really susceptible
    - Even
    - Quite susceptible
    - Very susceptible

10. **How likely is it that you will be diagnosed with one of the following medical conditions in the next 12 months?**

	Very unlikely	Unlikely	Even	Likely	Very likely
Seasonal influenza	○	○	○	○	○
Diabetes	○	○	○	○	○
Heart attack	○	○	○	○	○
Mexican flu	○	○	○	○	○
HIV or AIDS	○	○	○	○	○

11. **How likely is it that you will be diagnosed with one of the following medical conditions in the next 12 months, compared to others of your sex and age in the Netherlands?**

	Much less	Less	Same	More	Much more
Seasonal influenza	○	○	○	○	○
Diabetes	○	○	○	○	○
Heart attack	○	○	○	○	○
Mexican flu	○	○	○	○	○
HIV or AIDS	○	○	○	○	○

12. **Are you worried about the Mexican flu?**
    - Not at all worried
    - Not worried
    - A bit worried
    - Worried
    - Very worried

13. **Are you scared for the Mexican flu?**
    - Not at all scared
    - Not scared
    - A bit scared
    - Scared
    - Very scared

14. How often do you think about the Mexican flu?

- ○ Not at all
- ○ A view times
- ○ Often
- ○ Very often
- ○ I could not sleep

15. A number of preventive measures are mentioned below. For each measure, please indicate if you think <u>it will prevent you</u> from getting the Mexican flu.

	Certainly not	Probably not	Even	Probably	Certainly
Keep away from crowded places	○	○	○	○	○
Practice better hygiene	○	○	○	○	○
Avoid regions/persons with the flu	○	○	○	○	○
Wear face masks	○	○	○	○	○
Seek medical advice with the onset of symptoms	○	○	○	○	○
Take antiviral medication	○	○	○	○	○
Stay home from school or work	○	○	○	○	○
Get a new vaccine against the Mexican flu	○	○	○	○	○

16. Imagine that health authorities advice these measures. For each measure, please indicate if you think <u>you would be able</u> to take this measure.

	Certainly not	Probably not	Even	Probably	Certainly
Keep away from crowded places	○	○	○	○	○
Practice better hygiene	○	○	○	○	○
Avoid regions/persons with the flu	○	○	○	○	○
Wear face masks	○	○	○	○	○
Seek medical advice with the onset of symptoms	○	○	○	○	○
Take antiviral medication	○	○	○	○	○
Stay home from school or work	○	○	○	○	○
Get a new vaccine against the Mexican flu	○	○	○	○	○

17. Imagine that health authorities advice these measures. For each measure, please indicate <u>if you will take</u> this measure.

	Certainly not	Probably not	Even	Probably	Certainly
Keep away from crowded places	○	○	○	○	○
Practice better hygiene	○	○	○	○	○
Avoid regions/persons with the flu	○	○	○	○	○
Wear face masks	○	○	○	○	○
Seek medical advice with the onset of symptoms	○	○	○	○	○
Take antiviral medication	○	○	○	○	○
Stay home from school or work	○	○	○	○	○
Get a new vaccine against the Mexican flu	○	○	○	○	○

18. Below are statements, please indicate the degree to which you agree with each of these statements.

	Totally disagree	Mostly disagree	Don't disagree or agree	Mostly agree	Totally agree
There is nothing we can do about it	○	○	○	○	○
The threat is exaggerated by media and government	○	○	○	○	○
I will move to a place without influenza	○	○	○	○	○
I will stock up and stay indoors	○	○	○	○	○
It will not be as bad as predicted	○	○	○	○	○
We will all be completely powerless	○	○	○	○	○
We just have to accept it	○	○	○	○	○

19. What have you done so far to prevent yourself from getting the Mexican flu?

- Nothing
- I avoided crowded places
- I practiced better hygiene (washing hands more frequent, using tissues when coughing or sneezing)
- I bought a mouth mask
- I seek medical consultation
- I avoided persons with influenza like symptoms
- I bought antiviral medication (as Tamiflu)
- I got a vaccination against seasonal flu
- Other, namely..

20. Have you been vaccinated against seasonal flu during the last winter season (2008-2009)?

- Yes
- No
- Don't know

21. What is the amount of information you received about the Mexican flu?

- No information
- A little information
- Some information
- Much information
- Very much information

22. Where did you found the information about the Mexican flu?

- Newspapers
- Television
- Radio
- Internet
- Other, namely..

23. How much attention did you paid to the information about the Mexican flu?

- Very little
- Little
- Even
- Much
- Very much

24. Do you consider the information of the government on Mexican flu to be sufficient?

- Certainly not
- Probably not
- Even
- Probably
- Certainly

25. Do you consider the information of the government on Mexican flu to be reliable?

- Certainly not
- Probably not
- Even
- Probably
- Certainly

26. Which topic would you want to receive more information about?

- How the infection can be transmitted
- How the infection can be recognized
- Which protective measures can be taken to protect myself against infection
- The likelihood of infection
- How the infection can be treated
- Were I can get medication against the Mexican flu
- Other, namely..

27. Of whom would you like to receive this information? (multiple answers possible)

- o General practitioner
- o Local health institutes (i.e. Municipal Health Service)
- o National health institutes (i.e. National Institute of Public Health and the Environment)
- o Employer
- o Family/friends
- o Don’t know
- o Other, namely...

28. How would you like to receive this information?

- o Newspapers
- o Television
- o Radio
- o Internet
- o Don’t know
- o Other, namely..

This is the end of the survey. Thank you for cooperation. If you have any questions, don’t hesitate to contact us.
